# DNA topoisomerases: Advances in understanding of cellular roles and multi-protein complexes via structure-function analysis

**DOI:** 10.1002/bies.202000286

**Published:** 2021-01-22

**Authors:** Shannon J. McKie, Keir C. Neuman, Anthony Maxwell

**Affiliations:** 1 Department Biological Chemistry, John Innes Centre, Norwich, UK; 2 Laboratory of Single Molecule Biophysics, NHLBI, Bethesda, Maryland, USA

**Keywords:** antibiotics, anti-cancer drugs, DNA gyrase, DNA supercoiling, DNA topoisomerase

## Abstract

DNA topoisomerases, capable of manipulating DNA topology, are ubiquitous and indispensable for cellular survival due to the numerous roles they play during DNA metabolism. As we review here, current structural approaches have revealed unprecedented insights into the complex DNA-topoisomerase interaction and strand passage mechanism, helping to advance our understanding of their activities in vivo. This has been complemented by single-molecule techniques, which have facilitated the detailed dissection of the various topoisomerase reactions. Recent work has also revealed the importance of topoisomerase interactions with accessory proteins and other DNA-associated proteins, supporting the idea that they often function as part of multienzyme assemblies in vivo. In addition, novel topoisomerases have been identified and explored, such as topo VIII and Mini-A. These new findings are advancing our understanding of DNA-related processes and the vital functions topos fulfil, demonstrating their indispensability in virtually every aspect of DNA metabolism.

## Introduction

### DNA structure and topology have profound consequences for metabolism

**Figure 1:**
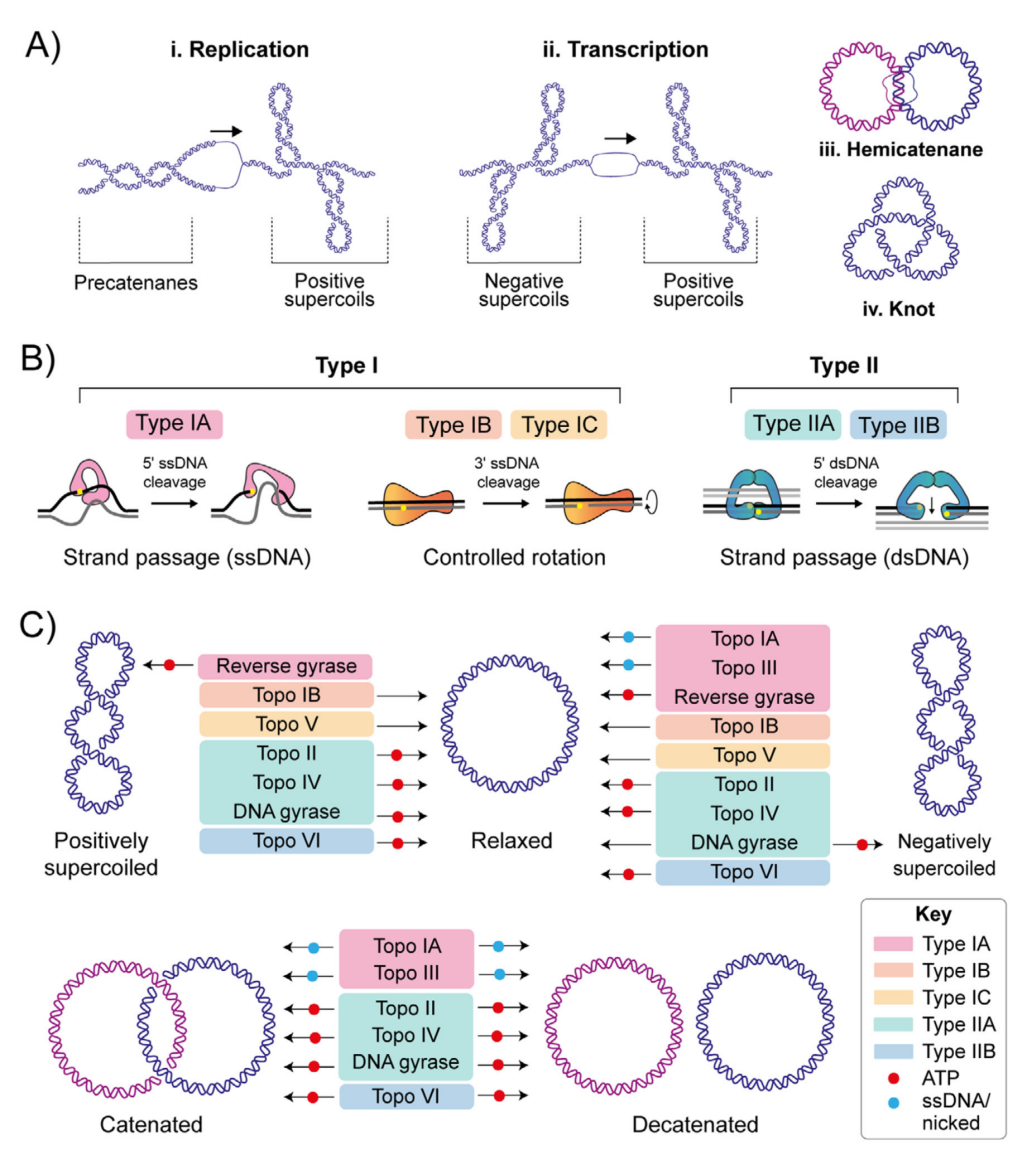
DNA topology and DNA topoisomerase mechanisms. (A) Topological consequences of DNA metabolism. i) During DNA replication, strand separation leads to positive supercoiling ahead of the advancing protein machinery, and precatenane formation behind. Precatenanes form as the newly-synthesised duplexes wrap around one and other, and, if not removed prior to complete of replication, catenated DNA molecules are formed. ii) During transcription, strand separation leads to positive supercoiling ahead of the advancing protein machinery, and negative supercoil formation behind. iii) Hemicatenanes are a possible end result of replication, in which the parental strands of the replicated duplexes remain base-paired. iv: DNA knotting can also occur as a result of DNA replication in which a DNA molecule is intramolecularly linked. (B) Summary of topo categories and mechanism. The topos are categorised based on whether they catalyse single- (type I) or double-stranded (type II) DNA breaks. The type I topos are further subdivided to type IA, IB and IC. Type IA form a transient covalent bond to the 5′ DNA phosphate and function via a strand passage mechanism. Type IB and IC form a transient covalent bond to the 3′ DNA phosphate and function via a controlled-rotation mechanism. Type II topos are further subdivided into type IIA and IIB. Both form a transient covalent bond to the 5′ DNA phosphate of both strands of the duplex and function via a strand-passage mechanism. (C) Summary of the topological manipulations performed by DNA topoisomerases, namely relaxation of positive and negative supercoils and decatenation. Type IA topos are colour-coded pink, type IB are orange, type IC are yellow, type IIA are green, and type IIB are blue. Requirement of ATP or ssDNA for activity is denoted using a red or blue circle, respectively

The DNA duplex is one of life’s fundamental molecules; therefore, maintaining its integrity is paramount. Potential topological issues associated with the double-helical structure were recognised soon after its structure was first elucidated in 1953 by James Watson, Francis Crick and Rosalind Franklin.^[1,2]^ The consequences of topological perturbations in DNA are exemplified by DNA replication during which the strands of the duplex are separated. This separation leads to the formation of positive supercoils (DNA overwinding) ahead of the replication fork and intertwining of the daughter strands, forming precatenanes, behind (Figure 1A).^[3,4]^ If the positive supercoils are not relaxed, progression of the replication fork is impeded, whereas failure to unlink the daughter strands prevents genome segregation, which is required for cell division.^[5]^ Transcription also generates positive supercoiling ahead of, and negative supercoiling behind, the transcriptional complex, known as the twin-supercoiled domain model, first described in 1987 (Figure 1A).^[6]^ These topological perturbations must be resolved for DNA metabolism to proceed, allowing the cell to efficiently replicate, transcribe and partition the genome to enable cellular division and vitality. However, in addition to the detrimental aspects of DNA topology that require resolution, beneficial aspects are harnessed by the cell to facilitate DNA melting and establish global genome architecture. For example, plasmid replication requires negative super-coiling of the origin, which facilitates local melting and exposes singlestranded DNA required for protein binding.^[7]^ Furthermore, compaction of the *E. coli* genome is achieved in part by significant negative super-coiling.^[8]^ The essential proteins responsible for performing these vital roles in controlling DNA topology are called the DNA topoisomerases (topos).

### Topos are structurally and mechanistically diverse

In general, all topos perform a similar task (i.e., interconverting the topological states of DNA), however, the precise ways in which this is achieved differs among enzyme classes (Figure 1B). A key feature linking all topos is the formation of a covalent DNA-topo intermediate in which the active site tyrosine of the topo forms a phosphotyrosyl linkage to the phosphate group in the DNA backbone via nucleophilic attack.^[4]^ Topos are classified as type I or type II depending on whether they catalyse the formation and re-ligation of single-stranded (ss) or double-stranded (ds)DNA breaks, respectively.^[4,9,10]^ The type I topos are further subcategorised as type IA, IB and IC. Type IA topos cleave the DNA backbone, generating a covalent linkage to the 5′-phosphate, in an Mg^2+^-dependent and ATP-independent manner (aside from reverse gyrase – see 2.1.3), and function via a strand passage mechanism.^[4,9,10]^ The type IB and IC topos cleave the DNA backbone, generating a covalent linkage to the 3′-phosphate (albeit using distinct active sites), independently of both ATP and Mg^2+^, and function via a controlled rotation mechanism.^[4,9,10]^ The type II topos are subcategorised as type IIA and IIB. Even though type IIA and IIB both catalyse dsDNA breaks through cleavage of the DNA backbone, generating a covalent linkage to the 5′-phosphate on both duplex strands, in an ATP/Mg^2+^ dependent manner, and function via a strand passage mech-anism (Figure 1B), they are structurally distinct.^[4,9,10]^ In addition, type IIA topos cause dsDNA breaks with 4-base overhangs, while type IIB generate 2-base overhangs. The structural and mechanistic differences throughout the topo family impart certain activity preferences, for example: preferential decatenation rather than relaxation (Figure 1C).

These intriguing enzymes have been of keen scientific interest since the first was discovered in 1971.^[11]^ As topos transiently disrupt the integrity of the DNA duplex in order to maintain it, they must function in a highly coordinated and precise manner to avoid generating permanent DNA breaks. Topos are important in human health and disease as this mechanism, along with their indispensability, makes them vulnerable to poisoning, which is exploited in the use and development of antimicrobial and anticancer therapeutics.^[12,13]^ Explored below are the numerous ways in which topoisomerases employ their DNA cleavage/re-ligation mechanism in the preservation of genome integrity, with a focus on new results pertaining to structure, mechanism, and in vivo roles. Advancing our understanding of this crucial protein family has led to significant insights into numerous DNA processing pathways, with it becoming clear that the activity of topos pervades essentially every aspect of DNA metabolism.

## Type Ia Dna Topoisomerases

### Topo I relaxes transcription-induced negative supercoiling

Prokaryotic DNA topoisomerase I (topo IA), initially isolated from *Escherichia coli*, is a 97 kDa monomer that relaxes negative supercoils.^[11]^ The main role in vivo for topo IA is thought to be preventing hyper-negative supercoil accumulation during transcription,^[14]^ which can disrupt DNA metabolism and genome integrity by promoting stable R-loop formation (a DNA:RNA hybrid) due to the increased probability of forming ss-DNA regions.^[15]^ Next-generation sequencing (NGS) techniques revealed that mycobacterial topo IA activity was highly correlated with RNA polymerase (RNAP) activity,^[16,17]^ and *E. coli* topo IA has been demonstrated to physically interact with the *β′* domain of RNAP via its CTD,^[18]^ localising it to transcription sites. Recently, mycobacterial topo I has also been shown to alter RNA topology and modify ribosomal RNA precursors,^[19]^ suggesting a potential RNA metabolism role in vivo. However, questions remain, including the detailed nature of the topo I and RNAP interaction in vivo, for example, does this only occur initially during topo I recruitment or do they remain bound throughout transcription, and what effect does this have on the processive removal of negative supercoils?

**Figure 2:**
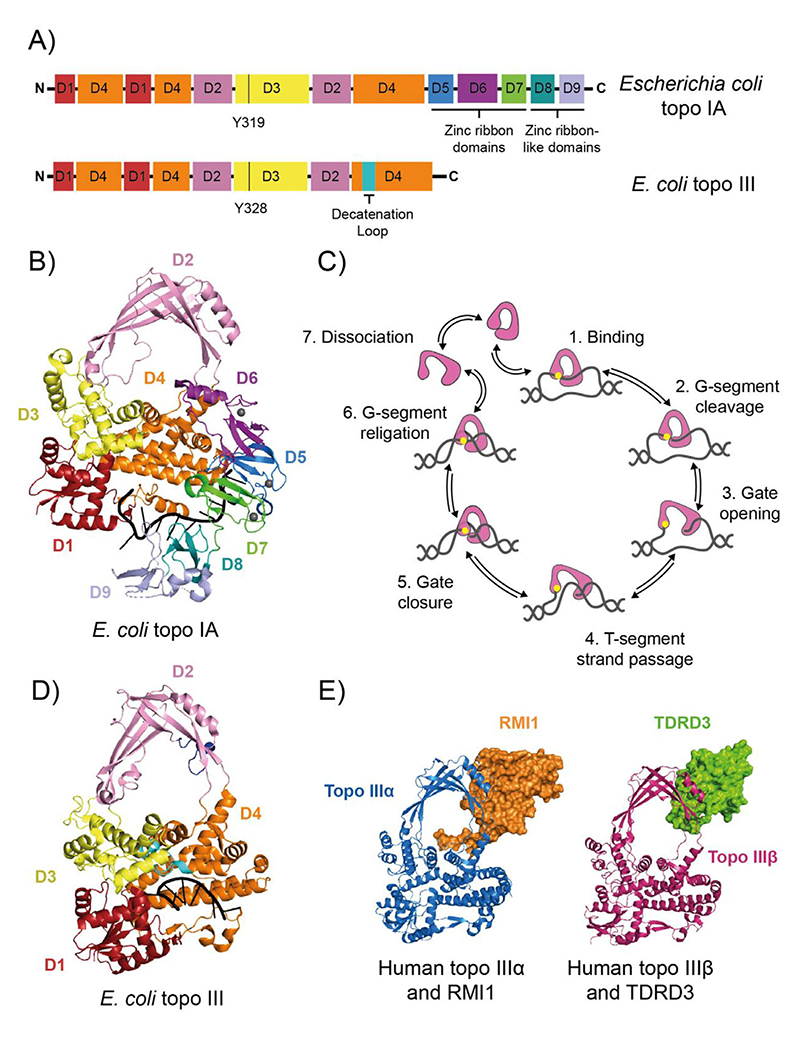
Type IA DNA topoisomerases. (A) Protein domain organisation of *Escherichia coli* DNA topoisomerase IA (topo IA) and DNA topoisomerase III (topo III). Black vertical lines represent the active site tyrosines. (B) Crystal structure of *E. coli* topo I bound to ssDNA (PDB: 4RUL).^[20]^ (C) Strand-passage mechanism for type IA topos. (1) topo binds G-segment ssDNA region, (2) the G-segment is cleaved. (3) The topo DNA-gate is opened, (4) which allows T-segment transfer through the cleaved G-strand. (5) The DNA gate is closed, (6) and the G-strand is re-ligated, changing the linking number by 1. (7) The topo can then go through another round of relaxation or dissociate from the DNA. Type IA topo (domains 1–4) is in pink, the active site tyrosine is yellow and the DNA is grey. (D) Crystal structure of *E. coli* topo III bound to ssDNA (PDB: 2O54).^[26]^ (E) Crystal structures of human topo III*α* (blue) bound to RMI1(orange) (PDB: 4CGY),^[39]^ and human topo III*β* (magenta) bound to TDRD3 (green) (PDB: 5GVE).^[60]^ For panels A, B and C, the topo I and III domains are colour coded as follows: D1 is red, D2 is pink, D3 is yellow, D4 is orange, D5 is marine blue, D6 is purple, D7 is green, D8 is teal, and D9 is light blue

Structural characterisation of full-length *E. coli* topo IA complexed with ssDNA revealed 9 distinct domains in a toroidal arrangement (Figure 2A,B).^[20]^ The N-terminal domains (NTDs) contribute to ssDNA binding and cleavage (domains 1, 3, 4), house the catalytic tyrosine (domain 3) and form a highly conserved hinge region with a flexible loop of charged residues, thought to play a role during strand passage (domain 2). The C-terminal domains (CTDs) include three 4-Cys zinc ribbon domains (domains 5–7) that interact with the DNA substrate, and two zinc ribbon-like domains (domains 8 and 9) that promote processivity by enhancing ssDNA binding.^[21]^ Intriguingly, recent work on *Helicobacter pylori* topo IA, has suggested that the CTD alone can catalyse DNA relaxation. This raises the heretical possibility of catalysis without the canonical active-site tyrosine. Whether this occurs in other species and whether there is an independent topo IB-like activity in the CTD, remain to be determined. ^[22]^

Based on the topo IA structure, a mechanistic model was proposed.^[20]^ A single DNA strand of the underwound duplex, known as the G (gate)-segment, is bound and cleaved at the 5' phosphate by the NTD, while the other strand, the T (transported)-segment, is bound by the CTD. The T-segment is then passed through the cleaved G-segment in a process termed strand passage, followed by G-segment re-ligation (Figure 2C). For strand passage to occur, topo IA must undergo a conformational change to open the DNA-gate and allow T-segment transfer. *E. coli* topo IA gate opening was observed directly using single-molecule magnetic tweezers, demonstrating that the DNA gate opens by 6.6 ± 1.0 nm and rapidly oscillates between open and closed conformations.^[23]^ This is hypothesised to reflect its in vivo role in the efficient and processive removal of negative supercoils. Recent structural characterisation of *Mycobacterium smegmatis* topo I has shown that the CTD can bind ssDNA with higher affinity than the NTD, suggesting it may bind the T-segment before the G-segment.^[24]^ This mechanism, coupled to the physical interaction with RNAP, makes topo I highly efficient in the relaxation of transcription-induced negative supercoiling, protecting genome integrity.

### DNA topoisomerase III

#### Topo III resolves interlinked replication intermediates

DNA topoisomerase III (topo III) is highly conserved across prokaryotes and eukaryotes.^[25]^ It closely resembles topo IA domains 1–4,^[26]^ but with two additional loops, amino acids 502–519 and 241–255 (*E. coli* numbering), the former important for decatenation activity, possibly through interaction with duplex DNA (Figure 2D).^[27]^ The mechanism is considered similar to topo IA (Figure 2C), but involves the intramolecular passage of a duplex T-segment, rather than a single DNA strand.^[25]^ Topo III is hypothesised to function primarily in decatenation pathways in vivo, efficiently resolving precatenanes in vitro,^[28]^ and recently shown to act at the *E. coli* replication fork in vivo, with topo III knock-outs markedly deficient in chromosome segregation.^[29]^ This work also demonstrated that topo III interacts with the DnaX complex of the DNA III polymerase holoenzyme, and in vitro, topo III precatenane resolution was significantly stimulated by the DnaX complex. This interaction likely localises topo III to precatenanes in vivo, and is analogous to the topo I-RNAP interaction during transcription. *E. coli* topo III also maintained an open DNA-gate for longer than topo I, potentially reflecting the role of topo III in the intermolecular passage of a duplex during decatenation.^[23]^ *E. coli* topo III is also known to cooperate with RecQ helicase and single-stranded DNA-binding protein in the resolution of stalled converging replication forks.^[30]^ In addition, *E. coli* topo III can perform strand passage on RNA, suggesting potential roles in resolving RNA topology.^[31,32]^

#### Topo III*α* is crucial member of DNA-repair complexes

In metazoal and some fungal species, topo III exists as two isoforms, topo III*α* and III*β*, which have been shown to play distinct roles in cellular development.^[25]^ Murine topo III*α* knockouts are embryo-lethal, demonstrating a fundamental role in preserving cellular viability.^[33]^ Human topo III*α* associates with BLM, a DNA repair-associated RecQ helicase,^[34]^ and RMI1 (or BLAP75), an oligonu-cleotide/oligosaccharide binding (OB) protein, forming a complex known as the dissolvasome.^[35]^ In human cells, RMI1 also interacts with RMI2 (or BLAP18), to form the RMI subcomplex, and RMI2 expression is interdependent on both RMI1 and topo III*α* expression.^[36]^ The dis-solvasome is integral to the non-crossover resolution of double Holliday junctions (dHJ),^[37]^ which are intermediates of the homologous recombination DNA-repair pathway.^[38]^ The structural characterisation of the RMI1/topo III*α* interaction revealed a 23-residue loop from RMI1 inserted into the topo III*α* cavity (Figure 2E), stabilising topo III*α* DNA-gate opening and promoting dHJ dissolution.^[39]^ The dissolva-some also interacts with FANCM (Fanconi anaemia group M protein), a DNA-repair protein that prevents the collapse of stalled replication forks.^[40]^ This supresses the ALT (alternative lengthening of telomeres) pathway,^[41]^ which is associated with DNA damage.^[42,43]^ Human topo III*α* cellular levels inversely correlated with tumour growth rate, with topo III*α* demonstrated to physically interact with the tumour suppressor protein, p53, and stimulate expression by binding the p53 promoter.^[44]^ In addition to modulating DNA-repair pathways, human topo III*α* was shown to interact with PICH DNA translocase (PIk1-interacting checkpoint helicase), generating positive supercoils via PICH-dependent loop extrusion of hypernegative supercoils that were relaxed by topo III*α*, potentially aiding in efficient centromere resolution.^[45]^

Topo III*α* is also localised to the mitochondria, and mutation of the mitochondrial import sequence in *Drosophila melanogaster* caused premature aging, mobility defects, and impaired fertility, caused by mitochondrial degeneration due to degradation of mitochondrial DNA (mtDNA).^[46,47]^ A Met100Val mutation in human topo III*α* was identified in a patient with a mitochondrial disorder and was demonstrated to prevent resolution of mtDNA replication-specific hemicatenanes (Figure 1A).^[48]^

#### Topo III*β* is involved in RNA metabolism

In contrast to topo III*α*, topo III*β* knockout mice survive, albeit with a reduced life span, development of autoimmune reactions, aneuploidy and infertility caused by accumulating chromosomal mutations.^[49–51]^ Recently in human cancer cells, the complete loss of topo III*β* caused genome instability due to increased R-loop formation, functionally link-ing topo III*β* to cancer suppression.^[52]^ Topo III*β* has also been shown to interact with RNA binding proteins (RBPs), TDRD3 (Tudor domain-containing 3) and FMRP (an RBP silenced in patients with Fragile X syndrome), localising topo III*β* to polyribosome-bound mRNA.^[53–56]^ Disruption of this interaction causes neurodevelopmental defects, suggesting that topo III*β* plays a distinct role during translation of specific mRNAs. Deletion or mutation of the topo III*β* gene is linked to schizophrenia and autism.^[53,57,58]^ In *Drosophila*, neuronal synapse formation was disrupted when an autism patient-derived mutation in topo III*β* was introduced.^[54]^ In addition, a disease-linked mutation in FMRP (I304N) from a Fragile X syndrome patient was shown to disrupt the interaction between FMRP and TDRD3/topo III*β*.^[55,59]^ The structure of the topo III*β*/TDRD3 complex reveals a largely hydrophobic inter-action between domain II of topo III*β* and the OB-fold of TDRD3 (Figure 2E), reminiscent of the topo III*α*/RMI1 interaction.^[39,60]^ However residues Arg96, Val109 and Phe139, along with the shorter TDRD3 insertion loop, were identified as crucial to the specific TDRD3/topo III*β* interaction.^[60]^ These recent results suggest that the main role of topo III*β* may be as an RNA topoisomerase, particularly crucial during neurodevelopment, and this has become an active and exciting area of research.

#### Reverse gyrase positively supercoils DNA in thermophiles

**Figure 3:**
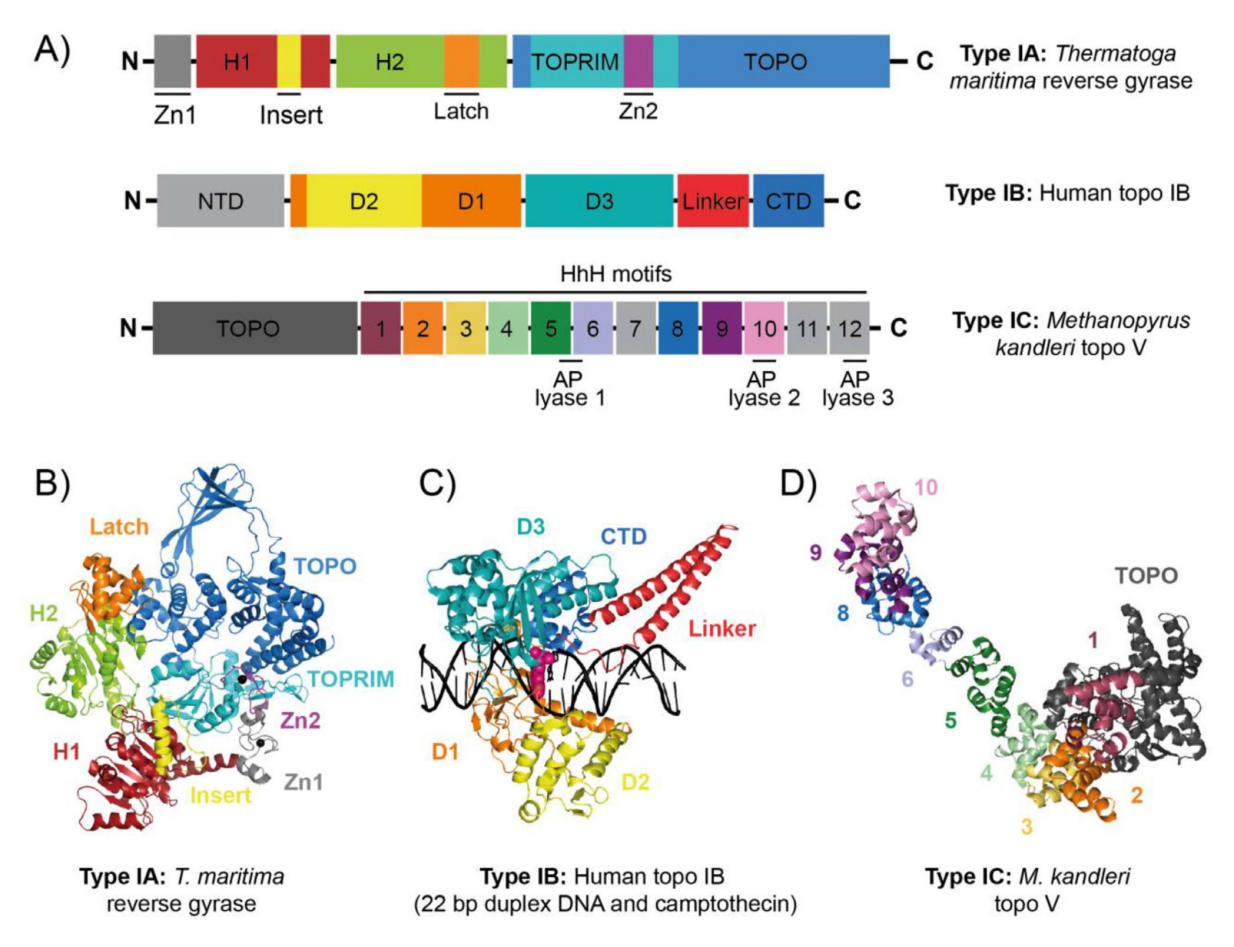
Reverse gyrase (type IA), topo IB (type IB) and topo V (type IC). (A) Protein domain organisation of *Thermatoga maritima* reverse gyrase, human DNA topoisomerase IB (topo IB), and *Methanopyrus kandleri* DNA topoisomerase V (topo V). (B) Crystal structure of *T. maritima* reverse gyrase (PDB: 4DDU).^[71]^ (C) Crystal structure of human topo IB in a cleavage complex with a 22 bp duplex DNA and camptothecin (PDB: 1T8I).^[220]^ (D) Crystal structure of *M. kandleri* topo V (PDB: 5HM5).^[89]^

Reverse gyrase, first discovered in *Sulfolobus acidocaldarius*, is a distinctive type IA topo that utilises ATP to introduce positive supercoils.^[61,62]^ It is found in thermophilic and hyperthermophillic archaea and eubacteria, thought to be important in preventing ther-mal DNA denaturation and aiding DNA-repair processes.^[63,64]^ Using magnetic tweezers, it was shown that *Sulfolobus tokodaii* reverse gyrase processively generates five positive supercoils s^–1^ on average,^[65]^ with loose coupling to ATP hydrolysis (20 s^–1^), consistent with previous measurements.^[66]^ Positive supercoiling by reverse gyrase is the combined activity of two distinct protein domains: the superfamily 2 helicase-like NTD and the topo IA-like CTD (Figure 3A,B).^[67]^

In the presence of ATP, the helicase domain of *Thermatoga maritima* reverse gyrase has high affinity for DNA and transiently destabilises the DNA duplex.^[68,69]^ In the absence of the helicase-like domain, the topo I-like domain has nucleotide-independent DNA relaxation activity,^[70]^ therefore the combination of DNA unwinding with strand passage permits positive supercoiling by reverse gyrase. The helicase and topo domains are coordinated via the latch domain, with bioinfor-matic analyses revealing significant latch sequence diversity amongst different reverse gyrases.^[71]^ In the absence of the *T. maritima* latch domain, reverse gyrase is unable to positively supercoil due to preventing DNA unwinding,^[69]^ although recently, a *β*-hairpin of the latch domain was demonstrated sufficient to maintain positive supercoiling activity.^[72]^

In addition to the reverse gyrase diversity among species, *Sulfolobus solfataricus* encodes two distinct copies of reverse gyrase, RG1 and RG2, which are alternatively regulated in vivo with separate biochemical activities.^[73–75]^ RG1 expression is sensitive to thermal stress, relaxing negatively-supercoiled DNA independently of ATP hydrolysis and distributively generating moderately-overwound DNA; whereas RG2 expression is constitutive and it processively generates highly-overwound DNA with a strict dependence on ATP hydrolysis. Magnetic tweezers assays demonstrated that DNA unwinding by RG2 was nucleotide-independent, while ATP hydrolysis was strictly required for strand passage.^[76]^ The physiological reasons for this variability in how reverse gyrases positively supercoil DNA remains to be determined.

## Type Ib Dna Topoisomerases

### Topo IB relieves torsional strain during transcription

The type IB topos, for example, eukaryotic DNA topoisomerase I (topo IB), were first discovered in 1972 and relax positive and negative supercoils via transient ssDNA cleavage of the DNA backbone, generating a covalent linkage to the 3’-phosphate.^[77,78]^ An N-terminally truncated 70-kDa human topo IB crystallised in complex with a 22-bp DNA duplex (Figure 3C) revealed how topo IB binds DNA and led to the proposal that it functions via a ‘controlled-rotation’ mechanism, first described for Vaccinia topo I.^[79,80]^ This involves topo IB creating a ssDNA nick, which permits DNA rotation of the free end around the intact strand, the speed controlled by friction within the enzyme cavity, before the nick is re-ligated.^[81]^ In vivo, topo IB is thought to relieve torsional strain in DNA, particularly during transcription.^[82]^ In line with this, the CTD of RNA polymerase II (RNAP II) is a potent activator of topo IB in vitro, and they have been shown to physically interact.^[83,84]^ The binding of topo IB strongly correlates with RNAP II binding in vivo at transcription start sites, and topo IB catalytic activity is observed in gene bodies, positively correlated with the level of gene expression.^[83]^ However, topo IB activity is also linked to transcription-associated mutations, characterised by 2–5 bp deletions in tandem repeats, particularly after topo IB cleavage at incorporated ribonucleotides.^[85]^ In addition, topo IB activity has been associated with numerous human diseases, including several spinocerebellar ataxia disorders and the autoimmune condition scleroderma.^[86]^ Inhibition of topo IB is used to suppress tumorigenesis and has also been shown to alleviate symptoms of Angelman syndrome (an autism spectrum disorder), potentially through preventing transcription of *UBE3A-ATS*, the RNA transcript of which causes pathogenesis.^[86]^

## Type Ic Dna Topoisomerases

### Topo V exhibits DNA relaxation and DNA repair activities

DNA topoisomerase V (topo V) was first isolated from the hyperther-mophillic methanogen, *Methanopyrus kandleri* and is the sole type IC member.^[87–89]^ Like type IB topos, topo V relaxes positive and negative supercoils without ATP and Mg^2+^, forming a covalent intermediate with the 3'-phosphate of the DNA, and functioning via a controlled-rotation mechanism. However, topo V was classed type IC as it was demonstrated to contain unique protein folds and an atypical active site, indicating an alternative cleavage/re-ligation mechanism.^[88]^ Topo V also exhibits DNA repair activity in vitro as an AP(apurinic or apyrimidinic)-lyase, potentially repairing abasic DNA damage.^[90,91]^ This DNA-repair activity functions independently of the topo activity as mutation of the active site tyrosine did not affect DNA-repair.^[90]^ Resolution of the 97-kDa topo V structure revealed a total of four active sites contained within a single polypeptide; one topo site and three AP lyase sites (Figure 3A).^[89]^ Topo V has a globular N-terminal topo-like domain, followed by 12 helix-hairpin-helix, motif 2 ((HhH)_2_) domains, which harbour the AP-lyase sites (Figure 3D). While fascinating, topo V only appears in *M. kandleri*, living within hydrothermal vents of the deep ocean, and has therefore been postulated to have a viral origin as it is unlikely that it arose de novo in the ancestral lineage of *M. kandleri*.^[92]^

## Type Iia Dna Topoisomerases

**Figure 4:**
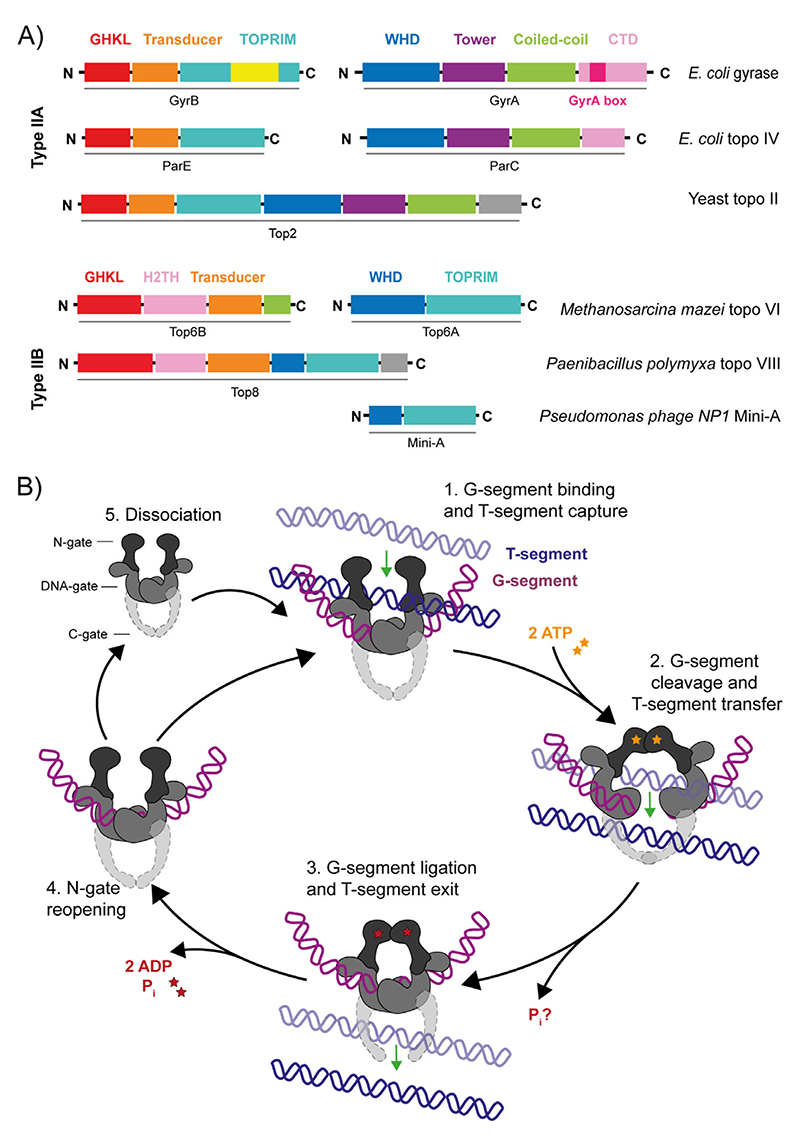
Type II DNA topoisomerases: domain organisation and mechanism. (A) Protein domain organisation for the type IIA topos: *E. coli* DNA gyrase, *E. coli* DNA topoisomerase IV (topo IV), yeast DNA topoisomerase II (topo II), *Methanosarcina mazei* DNA topoisomerase VI (topo VI), *Paenibacillus polymyxa* DNA topoisomerase VIII (plasmid-borne), and *Pseudomonas phage NP1* Mini-A. (B) type II topo strand passage mechanism. (1) G-segment is bound at the DNA-gate and the T-segment is captured. (2) ATP binding stimulates dimerisation of the N-gate, the G-segment is cleaved and the T-segment is passed through the break. (3) The G-segment is re-ligated and T-segment exits through the C-gate. For type IIB topos, there is no C-gate so once the T-segment passes through the G-segment, it is released from the enzyme. (4) Dissociation of ADP and P_i_ allows N-gate opening, a scenario where the enzyme either remains bound to the G-segment, ready to capture a consecutive T-segment, or (5) dissociates from the G-segment.

The type IIA topos include prokaryotic DNA gyrase (gyrase) and topoisomerase IV (topo IV), and eukaryotic topoisomerase II (topo II) (Figure 4A).^[10]^ The general mechanism for the type II topos begins with the binding of one DNA duplex, termed the gate segment (G-segment), at the DNA gate. Another duplex, termed the transport segment (T-segment), is captured by the ATP-operated clamp (N-gate) and passed through a transient break in the G-segment before it is released through the C-gate and the G-segment is re-ligated. The N-gate then reopens resetting the enzyme for another round of strand passage or release from the DNA (Figure 4B). The specifics of this reaction vary amongst type II topos, each being both intrinsically (e.g., structure) and extrinsically (e.g., protein-protein interactions and temporal/spatial regulation) adapted to preferentially perform different DNA topology manipulations. The formation and resealing of the DSB is highly efficient to prevent extensive genotoxic damage,^[93]^ but this also constitutes a juncture of vulnerability that is exploited by antimicrobial/anticancer drugs.^[12,94]^

The key protein domains shared amongst the type IIA topos and present in pairs within the holoenzyme, are the WHD (winged-helix domain, or 5Y-CAP), the TOPRIM (topoisomerase/primase) domain and the GHKL (DNA Gyrase, Hsp90, bacterial CheA-family histidine kinases and MutL) ATPase domain.^[95]^ The WHD contains a helix-turn-helix fold, commonly found in DNA-binding proteins, including the *E. coli* catabolite activator protein (CAP),^[96]^ and houses both the catalytic tyrosine residue, which forms a reversible covalent bond with the 5'-scissile DNA phosphate,^[97]^ and an isoleucine, which intercalates into the G-segment producing a ~150° bend,[98] promoting DNA cleavage.^[99]^

The TOPRIM domain chelates Mg^2+^ via the DxD motif, and contains a glutamate residue thought to act as a general acid during cleavage, donating a proton to the sugar hydroxyl, and a general base during religation, abstracting the proton from the 3′-OH.^[100,101]^ Together, the TOPRIM DxD motif and the active site tyrosine of the WHD, form a bipartite active site capable of cleaving the DNA backbone.^[95,102]^ The TOPRIM domain also contains conserved residues, namely the EGDS and PLRGK motifs, which interact with the G-segment and assist with DNA binding.^[102,103]^ The type IIA-specific tower domain also interacts with the G-segment as it exits the WHD, anchoring the outer portion of the bent duplex and promoting both DNA binding and cleavage efficiency.^[98]^

Recently, the way in which type II topos utilise divalent metal ions during DNA cleavage has been questioned.^[12]^ Early work supported the idea that each TOPRIM domain coordinated two Mg^2+^ ions in two pockets denoted sites “A” and “B,” with site A-bound Mg^2+^ participating in cleavage, and site B-bound Mg^2+^ anchoring the adjacent phosphate of DNA.^[104,105]^ However, a moving metal ion mechanism is now gaining support; that is, following cleavage, the metal bound at site A moves to site B (associated with protein and DNA conformational changes) where it cannot participate in cleavage/re-ligation chemistry and protects the tyrosyl-phosphate linkage during strand passage.^[12,106]^ However, confirmation of either model requires further structural and biochemical characterisation.

The GHKL ATPase domain binds ATP.^[95,107]^ The precise role(s) of ATP in type II topo activity is still unclear; however, it is hypothesised that free energy of ATP enables the formation of a stable protein-protein interface, protecting against the formation of genotoxic DSBs when the DNA gate is opened during strand passage.^[93]^ Explored below are the alternative ways in which the type IIA topos employ these protein domains to perform distinct roles in vivo.

### DNA gyrase negatively supercoils DNA

Gyrase, discovered in 1976, is a unique type IIA topo found predominantly in bacteria, but also present in plants, apicomplexans and archaea.^[108–112]^ Gyrase can introduce negative supercoils, relax positive supercoils and decatenate DNA in an Mg^2+^/ATP-dependent manner, and relax negative supercoils independently of nucleotide.^[10]^ *E. coli* gyrase is a 374-kDa heterotetramer formed from two GyrA (97 kDa) and two GyrB (90 kDa) subunits.^[113]^ As gyrase is essential for bacterial viability, and absent in humans, it has had significant and ongoing clinical success as an antibacterial target.^[114,115]^

It is thought that the fundamental role of gyrase in vivo is the introduction of negative supercoiling. Indeed, if gyrase is inhibited, the genome becomes relaxed, indicating that gyrase plays a role in the homeostatic maintenance of a negatively-supercoiled genome.^[116–119]^ Negative supercoiling is important for the initiation of DNA replication and transcription as underwinding the DNA promotes melting of the origin and gene promoters.^[120,121]^ In addition to this role, gyrase is also considered vital during the elongation phase of replication and transcription, relaxing positive supercoils ahead of the advancing protein machinery. This is supported by gyrase loss-of-function mutations causing a significant decline in replication and transcription.^[122]^ An in vitro DNA replication system demon-strated that gyrase preferentially removed positive supercoils ahead of the fork.^[123]^ Using NGS, the binding of mycobacterial gyrase was found to be enriched in areas of high transcriptional activity, directly correlated with the binding of RNA polymerase, and at the replication origin.^[16]^ Another NGS-based study on *E. coli* gyrase also found increased activity downstream of highly transcribed operons.^[124]^ Recent in vivo single-molecule imaging data suggest multiple gyrases (~12) cluster ahead of the DNA replication fork.^[125]^ This is supported by magnetic tweezers data demonstrating that multiple gyrases were recruited to highly overwound DNA.^[126]^

**Figure 5:**
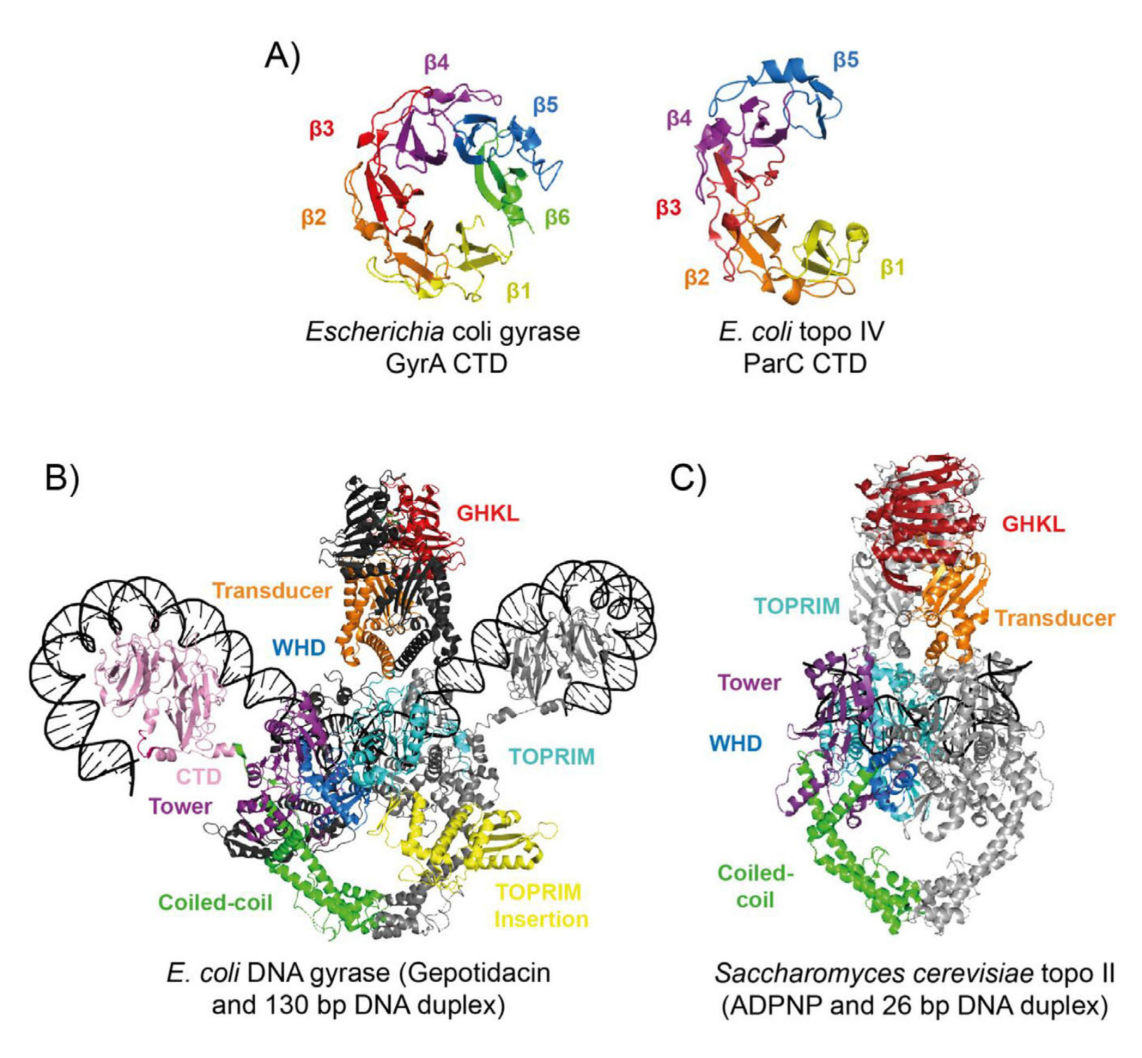
Type IIA DNA topoisomerase structures. (A) The *E. coli* gyrase GyrA CTD (PDB: 1ZI0)^[129]^ and the *E. coli* topo IV ParC CTD (PDB: 1ZVT).^[146]^ (B) CryoEM structure of full length *E. coli* gyrase complexed with a 130-bp DNA duplex and gepotidacin (PDB: 6RKW).^[137]^ Colour coding for domains is as labelled in the figure with the second GyrA and GyrB coloured light grey and dark grey, respectively, and the DNA in black. (C) Crystal structure of *Saccharomyces cerevisiae* topo II with a 26 bp DNA duplex and ADPNP (PDB: 4GFH).^[162]^ Colour coding of domains is as shown in the figure with second Top2 subunit coloured grey and the DNA in black

The unique negative supercoiling activity of gyrase arises from its capacity to wrap DNA via the CTDs of GyrA. The 35-kDa GyrA CTD has six *β*-strands in a *β*-pinwheel fold with a largely basic outer surface, indicating a role in DNA binding/bending, and the 7-residue GyrA-box (QRRGGKG), situated within a loop between *β*-strands 1 and 6, which is crucial for supercoiling (Figure 5A).^[127–132]^ Supercoiling is thought to begin with G-segment binding to gyrase and chirally wrapped around one of the GyrA CTDs, before being presented over the G-segment at ~60° as the T-segment, forming a left-handed (positive) crossing.^[133]^ Passage of the T-segment through the G-segment converts the crossing to a negative supercoil. Approximately ~130 bp of DNA is bound and wrapped by gyrase as measured by a variety of methods.^[134]^ Recent work using *Bacillus subtilis* gyrase with a single catalytic tyrosine suggested supercoiling activity could instead function via a nicking-closing mechanism, however this remains to be substantiated by other methods.^[135]^ Gyrase-DNA wrapping was recently demonstrated structurally using Cryogenic electron microscopy (cryo-EM), with a low-resolution (23 Å) structure of *Thermus thermophilus* gyrase in a cleavage complex with a 155 bp DNA duplex and ciprofloxacin, revealing asymmetric wrapping of DNA around the GyrA CTDs.^[136]^ In 2019, the first full-length cryo-EM structure of *E. coli* gyrase in complex with DNA and gepotidacin was solved, with DNA-binding domain resolution approaching 3.0 Å (Figure 5B). ^[137]^ This landmark structure provided an in-depth view of the overall architecture of DNA gyrase, revealing the spatial organisation of the domains, the position of the GyrA-box, and insight into DNA-cleavage site conformational changes, particularly in regard to the position of the TOPRIM insertion domain.

### Topo IV is critical for chromosome segregation in bacteria

Topo IV, discovered in *E. coli* in 1990, is a ~308 kDa heterotetramer composed of two ParC (~84 kDa) and two ParE (~70 kDa) subunits, which can relax positive or negative supercoils and decatenate, in an ATP/Mg^2+^-dependent manner (Figure 4A).^[138]^ The topo IV genes were discovered through DNA partitioning defects, which suggested that topo IV was involved in decatenation and chromosome segregation.^[139]^ This was supported by an in vitro replication system that demonstrated topo IV was highly efficient at unlinking replicated daughter chromosomes.^[140]^ Furthermore, using NGS, the binding/cleavage of topo IV was specifically enriched at the dif site, where *E. coli* chromosomes are unlinked.^[141]^ This work also demonstrated a physical interaction between XerCD recombinases, modulated by MatP, indicating that topo VI is part of a multi-protein system required for efficient chromosome segregation.^[141]^ MatP also regulates the physical interaction between topo IV and the *E. coli* SMC (structural maintenance of chromosomes) complex, MukBEF ^[142–145]^, at the origin of replication, enhancing topo IV decatenation.

In addition to protein-protein recruitment, the structure of topo IV also supports preferential decatenation activity. The topo IV ParC CTD has only five *β*-strands and no GyrA-box so does not permit supercoiling (Figure 5A). However, the outer surface is positively charged, suggesting a role in DNA binding and therefore potentially mediating topo IV substrate specificity.^[146]^ Indeed, deletion of the ParC CTD results in significant reductions in relaxation and decatenation rates in vitro, which is far more profound for the relaxation of positive supercoils and decatenation, than negative supercoil relaxation.^[146]^ Using single-molecule approaches, topo IV relaxed positive supercoils ~20-25-fold faster than negative, which has been attributed to the CTD of ParC stimulating high processivity during positive writhe relaxation, and suggests the topo IV CTDs recognise DNA geometries more common in positive supercoils and catenanes.^[146–149]^ There is evidence that topo IV can complement the activity of type IA topos, and support replication and transcription fork progression in vivo in cells encoding temperature-sensitive mutants of gyrase.^[150–153]^ However, topo IV seems to be a preferential decatenase in vivo through the combination of protein-protein recruitment, temporal regulation, and a structural preference for catenane geometries.^[141–146,154]^

### Yeast topo II is involved in DNA segregation, replication and transcription

Topo II is the major and essential type IIA topo found in eukaryotes. *Saccharomyces cerevisiae* (yeast) topo II is a homodimer (Figure 5A) that relaxes positive and negative supercoils, decatenates and unknots DNA, in an ATP- and Mg^2+^ -dependent manner.^[155]^ In vivo, the absence of yeast topo II prevented the completion of mitosis, suggesting a fundamental role resolving interlinked or knotted DNA, an activity which is promoted by the presence of condensin, an SMC complex.^[156–158]^ Yeast topo II also plays a role in the relief of torsional strain during DNA replication, with a preference for the relaxation of positive supercoils ahead of the fork.^[159,160]^ In addition, yeast topo II supports transcription of long genes (>3 kb), and its absence stalls fork progression, which cannot be rescued by topo I.^[161]^

The structure of yeast topo II (residues 408–1177) bound to a 30 bp G-segment, showed the DNA to be both A-form and bent to a ~150° angle, deformations thought to be important for DNA cleavage, correctly positioning the DNA backbone within the active site.^[98]^ In addition, a minimally-truncated, fully-functional yeast topo II structure (residues 1–1177) bound to DNA and ADPNP (Figure 5C), revealed that a loop of the transducer domain, named the K-loop, interacted with the G-segment.^[162]^ Mutagenesis within the K-loop didn’t affect DNA cleavage but caused a severe reduction in relaxation and decatenation activity for both yeast topo II and human topo II*α*, implicating the K-loop in strand passage.^[162]^

### Topo II*α* is essential for DNA replication and segregation

In vertebrates, topo II exists as two isoforms, topo II*α* and II*β*.^[163]^ Topo II*α* is critical for cellular viability, and has essential roles during DNA replication and mitosis, and a cell-cycle regulated expression pattern.^[163–165]^ It is well-established that topo II*α* is important for chromosome condensation,^[165–169]^ however, it has recently been shown to also be important for maintenance of chromosome structure, despite previous results suggesting the contrary.^[170,171]^ Chromatin compaction seems to arise, in part, from the interplay between topo II*α* and the SMC complexes, such as condensin.^[168]^ Topo II*α* is also integral to chromosome segregation, removing catenanes along the chromosome arms prior to the onset of metaphase,^[172,173]^ as well as at the centromere once cohesin has been removed by separase at the onset of anaphase.^[174]^ As the chromatids are pulled apart, interlinked DNA at the centromere forms ultra-fine anaphase bridges (UFBs) that are bound by PICH, stimulating the decatenation activity of topo II*α*.^[175]^ Topo II*α* is also involved in chromatid resolution at ribosomal DNA (rDNA) regions during anaphase alongside PICH, tankyrase and condensin II.^[175,176]^ In addition to the complex protein-protein interaction profile for topo II*α*, the CTD plays crucial roles in vivo, bearing a nuclear localisation signal,^[177]^ the chromatin tether domain (crucial for activity during mitosis),^[178]^ as well as sumoylation, acetylation, phosphorylation, and ubiquitination sites that regulate the activity of the enzyme in a cell-cycle-dependent manner.^[179,180]^

### Topo II*β* has crucial roles in neurodevelopment

Topo II*α* and II*β* have distinct roles in vivo, thought to be a consequence of the divergent CTDs imparting differential regulation and activity.^[181]^ Whereas murine topo II*α* knock-outs are embryonic lethal with expression restricted to proliferating cells, topo II*β* knock-outs die after birth due to respiratory failure and expression is detected in most adult tissues.^[182–184]^ Numerous studies have since implicated topo II*β* activity in neuronal development and transcription.^[181]^ Recently, activation of neuronal early-response gene expression, critical for external environment sensing, was linked to dsDNA break formation in the genes’ promoters, likely caused by topo II*β*.^[185]^ In addition, two patients with autism spectrum disorder and profound neurodevelopmental delays were reported to have a de novo heterozygous His58Tyr topo II*β* mutation, strongly suggesting that impairment of topo II*β* activity has a severe effect on brain development.^[186,187]^ In addition to its neurological role, topo II*β* has also been implicated in DNA repair, aging, HIV infection, and cancer,^[188]^ and like topo II*α*, the full extent of topo II*β*’s biological roles are beginning to be revealed.

In 2018, a 2.75-Å crystal structure of the open human topo II*β* DNA gate (residues 445–1201) revealed a fully opened G-segment, with no interactions between the separated DNA cleavage domains of the two subunits, and a channel large enough to allow passage of the T-segment. This has provided insight into the significant conformational changes type IIA topos undergo during strand passage.^[189]^

## Type Iib Dna Topoisomerases

### Topo VI is found in prokaryotes and eukaryotes

Topo VI, initially identified in the archaeal hyperthermophile *Sulfolobus shibatae*, has since been found throughout the archaea, a few bacterial species, and intriguingly, in eukaryotes such as plants and algae.^[190,191]^ Topo VI is a heterotetramer formed from two Top6A (~45 kDa) and two Top6B (~60 kDa) subunits, that relaxes positive and negative supercoils, and decatenates DNA.^[192]^ It is distinct from type IIA in terms of domain organisation, and having only two protein interfaces: the N-gate and the DNA-gate. Minimal homology to the type IIA topos is mainly found in the WHD, TOPRIM and GHKL domains (Figure 4A).^[193,194]^ This simplicity in terms of structure has made topo VI of keen interest in the dissection of the type II mechanism, particularly the role of ATP in the opening/closing of the N-gate.^[194]^

**Figure 6:**
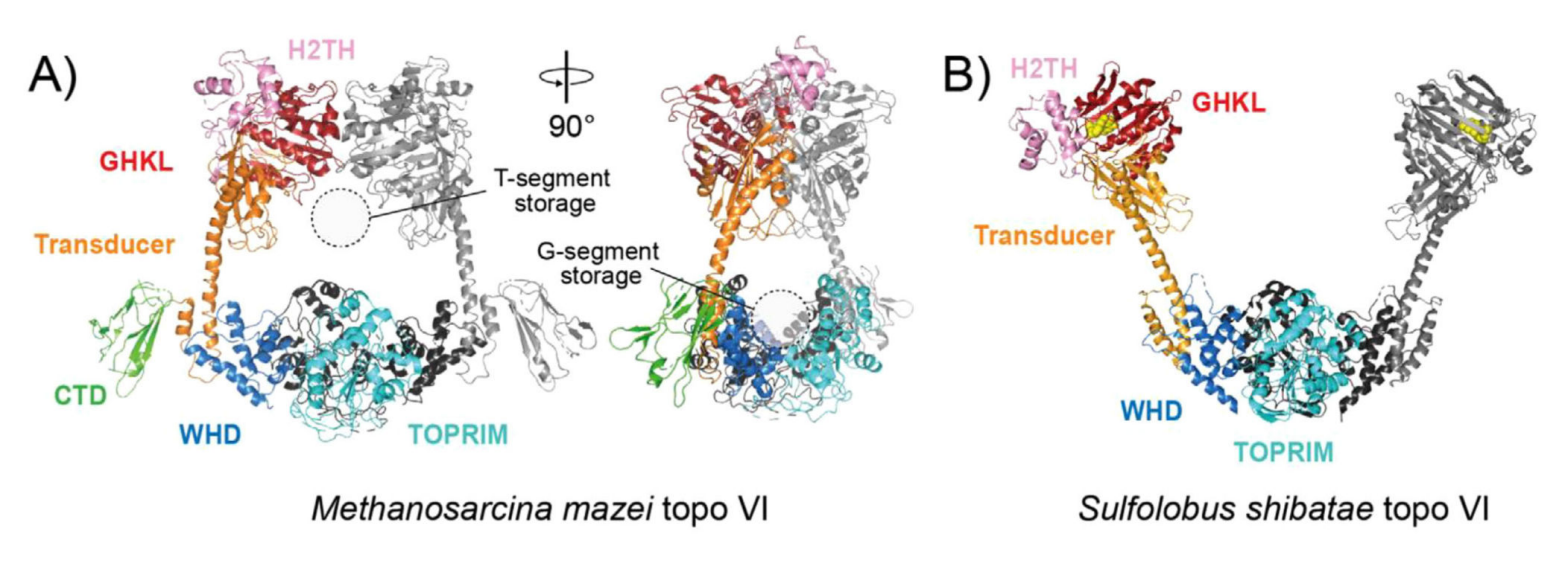
DNA topoisomerase VI (type IIB) structures. (A) Crystal structure of *Methanosarcina mazei* topo VI (PDB: 2Q2E).^[196]^ The domains are coloured as labelled in the figure on one TOP6A/Top6B heterodimer, with the second Top6A and Top6B coloured black and grey, respectively. (B) Crystal structure of *Sulfolobus shibatae* topo VI bound to radicicol (PDB: 2ZBK).^[197]^ Colour coding is the same as in panel A except GHKL-bound radicicol is coloured yellow

All topo VI structural characterisation has been performed using the archaeal forms, and includes both Top6A and Top6B independently, as well as the full-length heterotetramer in a “closed” (Figure 6A) and “open” conformation (Figure 6B).^[193–197]^ Together, these structures have revealed a clamp-like arrangement, with the Top6A dimer forming a positive electrostatic groove capable of accommodating the G-segment.^[193]^ The Top6B dimer forms a cavity large enough for a DNA duplex, which has recently been shown biochemically to be crucial for T-segment sensing and tightly coupling Top6B ATPase activity to strand scission by Top6A.^[198]^ Structural characterisation of archaeal Top6B in complex with ATP and hydrolysis-product analogues, allowed the ATP-mediated strand-passage mechanism to be modelled in more detail.^[193,194,196]^ This revealed that the transducer domain alternates between a “restrained” and “relaxed” state mediated by the respective association and dissociation of a conserved lysine residue with the *γ*-phosphate of the bound nucleotide. This transducer domain movement is thought to be coupled to strand scission and DNA-gate opening. As Top6B and *E. coli* gyrase GyrB share highly conserved motifs, this mechanistic insight likely applies to type IIA topos.^[194]^

Topo6A and Top6B homologues have been identified in plants such as *Arabidopsis thaliana*, named AtSPO11-3 and AtTOP6B, respectively.^[199,200]^ Homozygous knockouts of AtSPO11-3 or AtTOP6B are associated with growth-stunted phenotypes, which were demonstrated to be caused by endoreduplication defects. Endoreduplication facilitates cellular enlargement through multiple rounds of genome replication in the absence of cellular division.^[191,201,202]^ Why *A. thaliana* requires topo VI exclusively during endoreduplication remains unclear. Hypotheses include the resolution of endoreduplication-specific DNA structures, or endoreduplication-specific expression.^[203]^

Plant topo VI uniquely interacts with two accessory proteins named BIN4 (brassinosteroid-insensitive4) and RHL1 (roothairless-1), with mutants in either of these proteins also causing endoreduplication defects.^[204,205]^ The basis of the interaction between BIN4, RHL1 and topo VI is still unknown. The in vivo roles for plant topo VI have recently been expanded, with the discovery of interactions with plant steroid hormone genes; a role in chromatin organisation and transcriptional silencing, via interaction with the MIDGET protein through RHL1; abscisic acid (ABA) resistance, high salt tolerance, dehydration resistance; and reactive oxygen-species response.^[206–210]^ This demon-strates extensive roles for topo VI in mediating the plant’s response to endogenous and exogenous cues, integrating them through chromatin remodelling and transcriptional control.

Soon after the discovery of topo VI it was found that Top6A was highly homologous to the eukaryotic recombination factor, Spo11, which is responsible for the formation of dsDNA breaks during meiosis.^[211,212]^ Recently, Top6B structural homologues have also been identified that interact with Spo11 in mouse and *Arabidopsis thaliana*, to form the Spo11 complex.^[213,214]^ These important findings highlighted the evolutionary connection between topo VI and the meiotic machinery, and while Spo11 cannot reseal DNA cleavage, its structural similarity to topo VI suggests that the Spo11 complex may function in a similar manner.

There is uncertainty about whether a bone fide topo VI exists in plasmodia, as one study annotated *Top6A* and *Top6B* in the genome of *Plasmodium falciparum* ^[215]^, whereas, subsequent analysis concluded there was a *Spo11*, but no *Top6A*.^[216]^ This was further confounded by the result that topo VI from *P. falciparum* complemented the function of topo II in *S. cerevisiae* when expressed transiently.^[217]^ If topo VI is present in plasmodia, it is hypothesised to play a role in asexual reproduction and could have promise as a novel antimalarial drug target,^[215]^ but more work is required.

### Topo VIII is a novel type IIB topo

DNA topoisomerase VIII (topo VIII), discovered in 2014, is a novel member of the topo family. Identified through database screening using *S. shibatae* Top6B, topo VIII was classified as a highly divergent type IIB.^[218]^ Currently, 77 topo VIII enzymes have been identified in nine bacterial phyla, four in archaea (euryarchaeota phylum), and one unclassified.^[219]^ Topo VIII is distinct from topo VI as it is more common in bacteria than archaea, exhibits dramatic sequence divergence, is usually a homodimer, and encoding is dependent on plasmids and integrated elements.^[219]^ In addition, distantly-related Mini-A proteins were also identified in archaeoviruses and bacteriophages as truncated homologues of Top6A (Figure 4A).^[219]^

Of the topo VIII enzymes currently characterised biochemically, two exhibited Mg^2+^-dependent relaxation of positive and negative supercoils (*Microscilla marina* and *Paenibacillus polymyxa*), however the *M. marina* topo VIII performed these reactions independent of ATP, a behaviour not typical of type IIB topos.^[218]^ Topo VIII from *Ammonifex degensii* only demonstrated ATP-independent cleavage activity. In each case the activity was weak, moreover, many of the topo VIII enzymes appear to be in variable states of inactivation, so whether topo VIII plays considerable in vivo roles remains unclear.^[219]^

## Conclusions and Future Directions

Since the remarkable discoveries of bacterial topo I and DNA gyrase, topos have taken centre-stage in a wide range of metabolic DNA processes beyond their crucial roles in replication and transcription.^[11,108]^ It is becoming clear that topos play integral roles at multiple scales in the repair, segregation, and global organisation of nucleic acids. Continuing advances in cell biology and NGS approaches, that both provide spatial and temporal maps of protein activity, will undoubtedly expand and clarify our understanding of the central roles played by topos in many metabolic DNA processes. Complementing this, recent structural data has provided unprecedented insight into topo structure and function. These have included the full length DNA gyrase cryo-EM structure in complex with DNA, demonstrating the DNA wrapping behaviour; the open DNA gate of human topo II, informing further on the strand passage mechanism; and the *M. smegmatis* topo IA structure, indicating the T-strand may be bound prior to the G-strand.^[24,136,137,189]^ The use of single-molecule technologies, such as magnetic tweezers, have revealed aspects of topo activity previously unseen, including the gate opening dynamics of *E. coli* topo I and III, and the detailed dissection of the *S. solfataricus* RG2 reaction mechanism.^[23,76]^ It has also become clear that many topos form molecular complexes with other proteins that operate in a concerted way to maintain genome integrity, including the interactions of topo II*α* and topo III*α* with PICH, and topo III*β* with the RBPs, TDRD3 and FMRP.^[45,53–56,175]^ Despite 50 years having passed since topos were first discovered, the true extent and intricacy of their activities are still being enthusiastically explored and expanded, further consolidating the significance of topos in cellular viability.

## Data Availability

Data sharing is not applicable to this article as no new data were created or analyzed in this study.
